# Supplementary material for: A self-healing catalyst for electrocatalytic and photoelectrochemical oxygen evolution in highly alkaline conditions
Source: Nat Commun. 2021 Oct 13;12:5980. doi: 10.1038/s41467-021-26281-0 (PMC8514436; doi:10.1038/s41467-021-26281-0)
Supplement: Supplementary file 1 — Supplementary Information [file 41467_2021_26281_MOESM1_ESM.pdf]

Supplementary Information

A self-healing catalyst for electrocatalytic and photoelectrochemical oxygen evolution in highly alkaline conditions

Chao Feng, Faze Wang, Zhi Liu, Mamiko Nakabayashi, Yequan Xiao, Qiugui Zeng, Jie Fu, Qianbao Wu, Chunhua Cui, Yifan Han, Naoya Shibata, Kazunari Domen, Ian D. Sharp, Yanbo Li

Supplementary Methods

1. In-situ EQCM measurement of the deposition rates of Fe hydroxides.

The 0.25 M KB_i electrolyte (pH 14) containing 50 μM Fe(II) ions was prepared and aged in air for approximately 100 h. The deposition rates of Fe hydroxides in the electrolyte at room temperature under different applied potentials were measured by EQCM. The in-situ electrodeposition of Fe hydroxides was performed in a three-electrode configuration using Ti/Au QCM sensors as the working electrodes, an Ag/AgCl reference electrode, and a Pt counter electrode. The potential of the working electrode was controlled with a potentiostat (CHI600E) and varied in the range of 1.54-1.76 V vs. RHE. The mass changes of the QCM sensors before and after applying the potentials for 5 min were recorded from the QCM digital controller (Stanford Research Systems, QCM200), from which the deposition rates of Fe hydroxides were calculated. It should be noted that the EQCM system has a typical noise of ±3 ng cm^{-2}. Below the applied potential of 1.62 V vs. RHE, the amount of Fe hydroxides deposited on the QCM sensors was below the detection limit.

2. Determination of Fe(II) concentration in the electrolyte by colorimetric method.

The determination of Fe(II) concentration in the presence of Fe(III) in the electrolyte is made possible using a colorimetric method developed by Stumm et al. and widely used in the field of water chemistry[1]. It was found that ferrous iron (Fe(II)) forms strongly red-colored complexes with bathophenanthroline while ferric iron (Fe(III)) forms only slightly colored complexes with bathophenanthroline. The determination of low Fe(II) concentration in the presence of high Fe(III) background is therefore possible with this method.

2.1. Preparation of reagents.

1) *Ethyl alcohol* (C_2H_6O, anhydrous, AR, 99.7%) was purchased from MREDA. It was further purified by vaporization and condensation before use.

2) *1-Hexanol* ($C_6H_{14}O$, 99%) was purchased from Aladdin. It was further purified by vaporization and condensation before use.

3) *Hydrochloric acid* (HCl, 38%) was purchased from Chron Chemicals. It was diluted into 2 M using de-ionized water.

4) *Bathophenanthroline* (4,7-diphenyl-1,10-phenanthroline, $C_{24}H_{16}N_2$, 99%) was purchased from Aladdin. 1 mM stock solution was by dissolving 0.0332 g bathophenanthroline in 50 ml purified ethyl alcohol and diluting with 50 mL de-ionized water.

5) *Sodium acetate* ($C_2H_3NaO_2$, anhydrous, 99%) was purchased from Aladdin. 1.2 M iron-free stock solution was prepared by dissolving 9.9430 g sodium acetate in 100 mL de-ionized water in a 125-mL separatory funnel. 2 mL bathophenanthroline solution (1 mM) was added into the solution and well mixed. 10 mL purified 1-hexanol was then added and the mixed solution in the separatory funnel was shaken vigorously. After clear separation of liquids into two layers, the lower aqueous layer was drawn off into a glass-stoppered reagent bottle.

6) *Hydroxylammonium chloride* ($H_2NOH·HCl$, GR, 99%) was purchased from Aladdin. 1.4 M iron-free stock solution was prepared by dissolving 9.8268 g hydroxylammonium chloride in 100 mL de-ionized water in a 125-mL separatory funnel. 2 mL bathophenanthroline solution (1 mM) was added into the solution and well mixed. 10 mL purified 1-Hexanol was then added and the mixed solution in the separatory funnel was shaken vigorously. After clear separation of liquids into two layers, the lower aqueous layer was drawn off into a glass-stoppered reagent bottle.

2.2. Calibration curves for Fe(II) and Fe(III) concentrations in KB_i solution

1) *Calibration curve for Fe(II) concentrations.* 10 mM $FeSO_4$ solution was prepared with de-ionized water. 15 mL KB_i solution (0.25 M, pH 14) was added in a 125-mL separatory funnel. Different amount of $FeSO_4$ solution (3-75 μl) was pipetted into the KB_i solution to achieve different Fe concentrations (2-50 μM). The pH of the solution was adjusted to ~1.5 by adding the 2 M HCl solution. Because the Fe(II) ions added to the solution could be self-oxygenated into Fe(III), 2 mL iron-free hydroxylammonium chloride (1.4 M) solution was added to the solution to reduce the Fe(III) back to Fe(II). 15 mL bathophenanthroline solution (1 mM) was added and well mixed. The pH of the solution was adjusted to ~3.0 by adding the 1.2 M iron-free sodium acetate solution. 10 mL purified 1-hexanol was then added and the mixture was shaken thoroughly. The liquids

were allowed to separate for at least 5 min after shaking. After the liquids had cleanly separated into two layers, the lower aqueous layer was drawn off and discarded. The upper layer was drained into a 25-mL volumetric flask. The wall of the separation funnel was washed uniformly with 2-3 mL purified ethyl alcohol 3 times. The washes were transferred into the volumetric flask. The solution in the flask was diluted into 25 mL with purified ethyl alcohol and mixed by shaking. The absorbance of the solution in a 10-mm quartz glass cuvette was measured using a UV-Vis spectrophotometer (Shimadzu UV-2600). The absorbance of the solutions at 533 nm was then plotted against the Fe(II) concentrations. As shown in Supplementary Fig. S11, the color of the Fe(II)-bathophenanthroline conforms to Beer's law, so that a plot of the absorbance against the Fe(II) concentration yields a straight line with the slope equal to the absorptivity.

2) *Calibration curve for Fe(III) concentrations.* 10 mM $Fe_2(SO_4)_3$ solution was prepared with de-ionized water. 15 mL KB_i solution (0.25 M, pH 14) was added in a 125-mL separatory funnel. Different amount of $Fe_2(SO_4)_3$ solution (45-90 μl) was pipetted into the KB_i solution to achieve different Fe concentrations (30-60 μM). The pH of the solution was adjusted to ~1.5 by adding the 2 M HCl solution. 15 mL bathophenanthroline solution (1 mM) was added and well mixed. The pH of the solution was adjusted to ~3.0 by adding the 1.2 M iron-free sodium acetate solution. 10 mL purified 1-hexanol was then added and the mixture was shaken thoroughly. The liquids were allowed to separate for at least 5 min after shaking. After the liquids had cleanly separated into two layers, the lower aqueous layer was drawn off and discarded. The upper layer was drained into a 25-mL volumetric flask. The wall of the separation funnel was washed uniformly with 2-3 mL purified ethyl alcohol 3 times. The washes were transferred into the volumetric flask. The solution in the flask was diluted into 25 mL with purified ethyl alcohol and mixed by shaking. The absorbance of the solution in a 10-mm quartz glass cuvette was measured using a UV-Vis spectrophotometer. The absorbance of the solutions at 533 nm was then plotted against the Fe(III) concentrations (Supplementary Fig. S11). The absorbance of the Fe(III)-bathophenanthroline solution is more than 20 times lower than that of the Fe(II)-bathophenanthroline solution.

2.3. Determination of Fe(II) concentrations in the KB_i electrolyte during OER test.

At different times during the 100-h chronoamperometry test at 10 mA cm^{-2}, aliquots (15 mL) of the 50 μM Fe-containing KB_i electrolyte were taken and added in a 125-mL separatory funnel. The pH of the solution was adjusted to ~1.5 by adding the 2 M HCl solution. 15 mL bathophenanthroline solution (1 mM) was added and well mixed. The pH of the solution was adjusted to ~3.0 by adding the 1.2 M iron-free sodium acetate solution. 10 mL purified 1-hexanol was then added and the mixture was shaken thoroughly. The liquids were allowed to separate for at least 5 min after shaking. After the liquids had cleanly separated into two layers, the lower aqueous layer was drawn off and discarded. The upper layer was drained into a 25-mL volumetric flask. The wall of the separation funnel was washed uniformly with 2-3 mL purified ethyl alcohol 3 times. The washes were transferred into the volumetric flask. The solution in the flask was diluted into 25 mL with purified ethyl alcohol and mixed by shaking. The absorbance of the solution in a 10-mm quartz glass cuvette was measured using a UV-Vis spectrophotometer (Supplementary Fig. S12). The concentrations of Fe(II) in the electrolyte during the OER test were calculated from the absorbance of the samples at 533 nm and the calibration curve for Fe(II) concentrations after subtracting the background absorbance of Fe(III)-bathophenanthroline complexes.

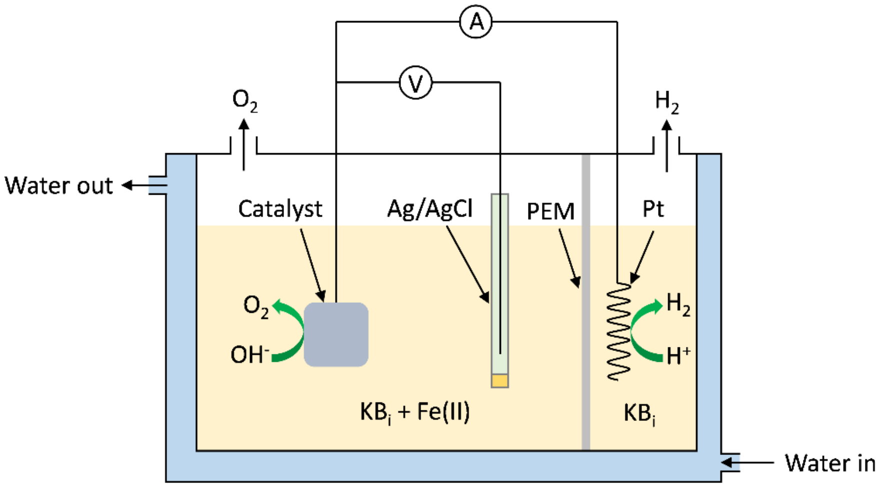

Supplementary Fig. S1 | Schematic drawing of the three-electrode electrochemical cell.

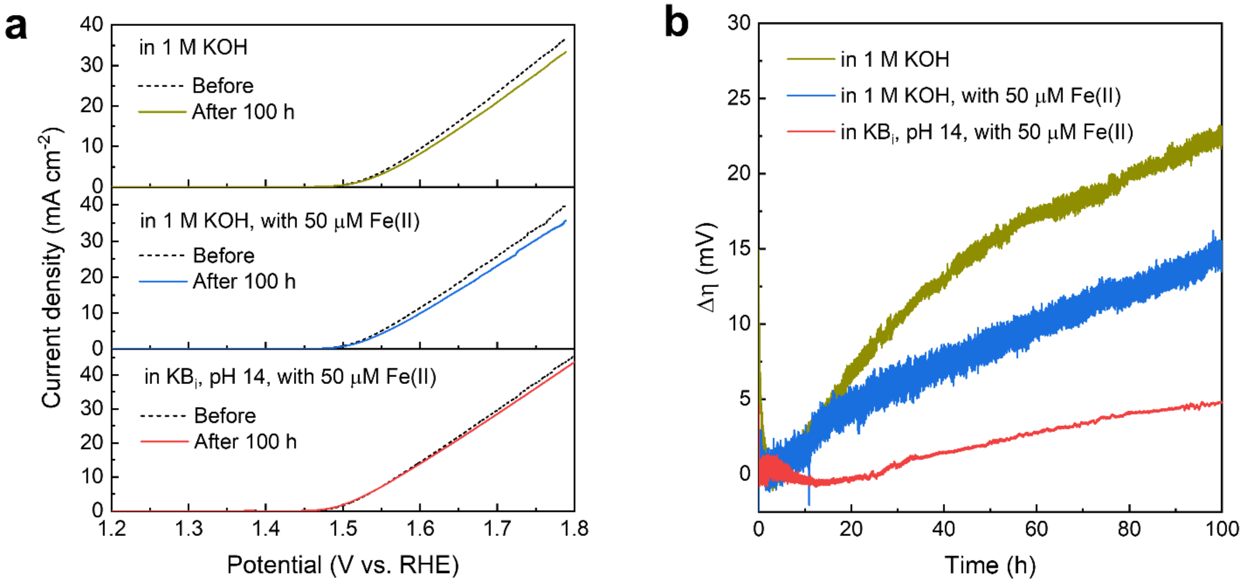

Supplementary Fig. S2 | Stability of the NiFe-B_i catalysts in diffrerent electrolyte. a, The OER polarization curves of NiFe-B_i catalysts before and after stability test for 100 h in different electrolytes. The curves were measured at 1 mV s^{-1} scan rate, without iR correction. **b,** Change of overpotentials (η) for NiFe-B_i catalysts measured at constant current density of 10 mA cm^{-2} for 100 h in different electrolytes.

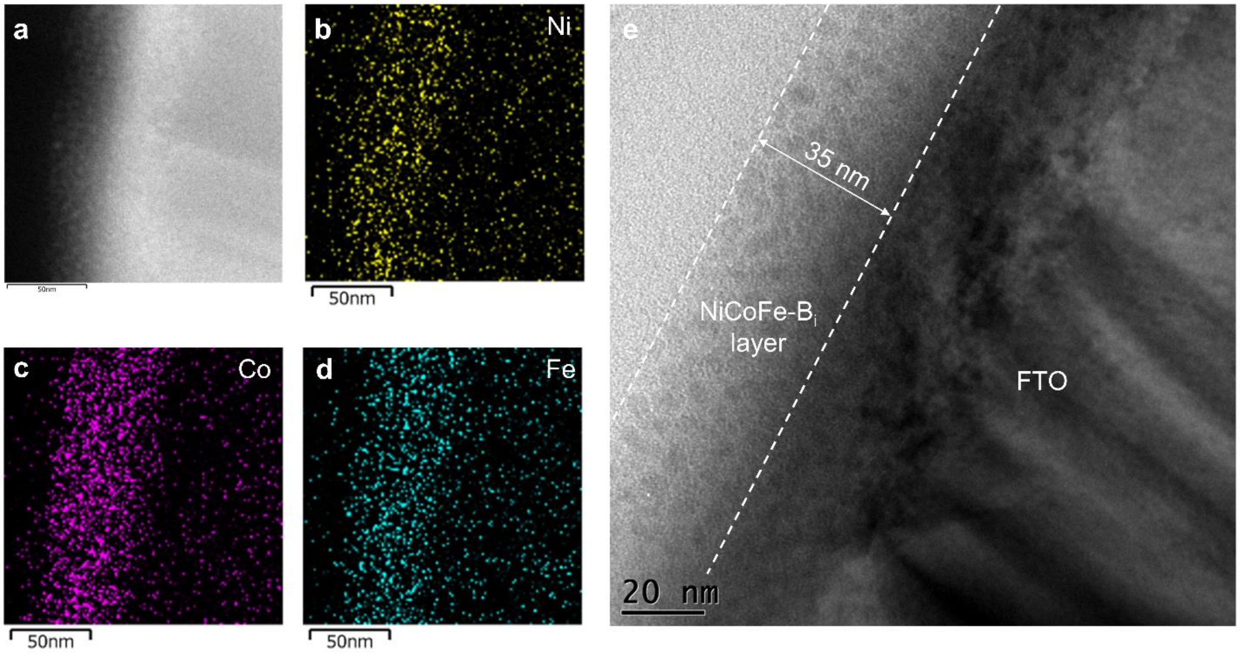

Supplementary Fig. S3 | Structural properties of NiCoFe-B$_i$ catalyst electrodeposited on FTO substrate. a, Scanning transmission electron microscopy (STEM) image of a NiCoFe-B$_i$ layer on FTO substrate and corresponding energy-dispersive X-ray spectroscopy (EDS) mappings of **b**, Ni, **c**, Co, and **d**, Fe. **e**, High-resolution transmission electron microscopy (HRTEM) image of the NiCoFe-B$_i$ layer on FTO substrate. The thickness of the NiCoFe-B$_i$ layer is approximately 35 nm.

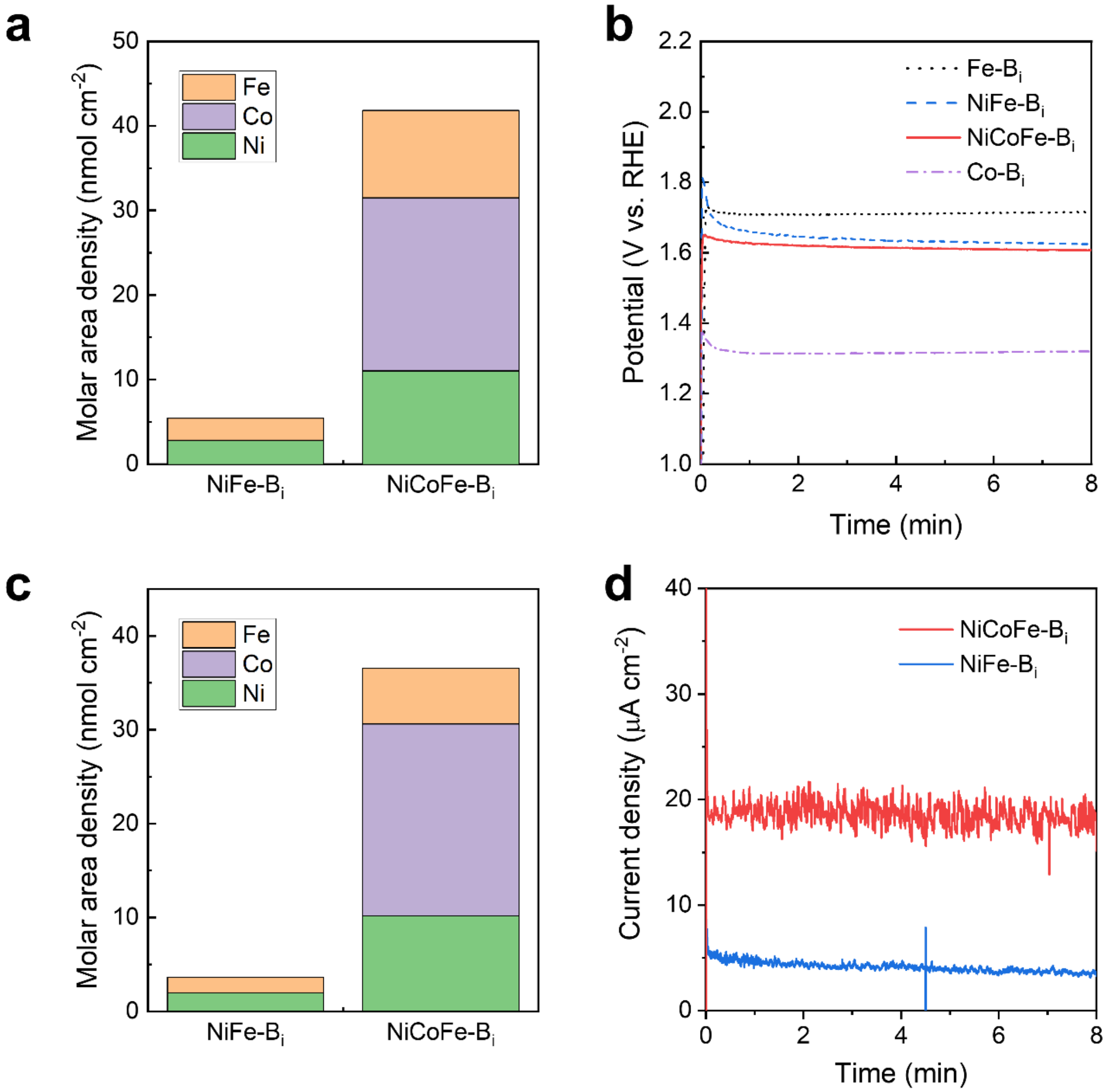

Supplementary Fig. S4 | Catalytic effect of Co on the deposition of Fe and Ni. a, ICP-MS measured molar surface density of the NiFe-B_i and NiCoFe-B_i catalysts electrodeposited on FTO substrates at the same current density of 20 μA cm^{-2} for 8 min. **b,** Chronopotentiometry curves for the electrodeposition of NiFe-B_i, NiCoFe-B_i, Fe-B_i, and Co-B_i catalysts at 20 μA cm^{-2}. **c**, ICP-MS measured molar surface density of the NiFe-B_i and NiCoFe-B_i catalysts electrodeposited on FTO substrates at the same applied potential of 1.6 V vs. RHE for 8 min. **d**, Chronoamperometry curves for the electrodeposition of NiFe-B_i and NiCoFe-B_i catalysts at 1.6 V vs. RHE. With the presence of Co(II) ions in the electrolyte, the amounts of Fe and Ni deposited on the substrate were significantly increased either at the same deposition current density (**a**) or at the same deposition potential (**c**).

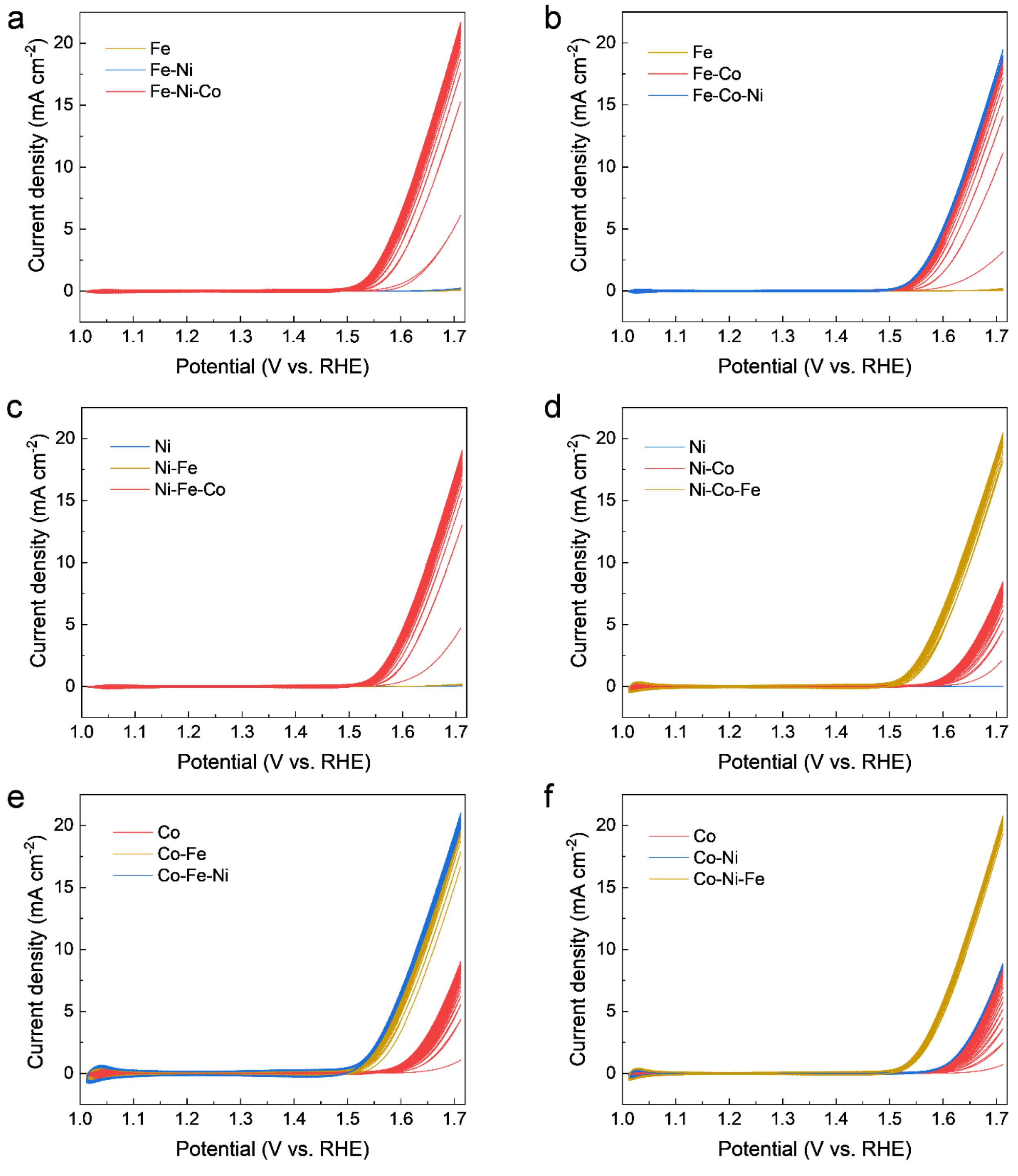

Supplementary Fig. S5 | In-situ sequential deposition of Ni, Co, and Fe oxyhydroxides on FTO substrates. a-f, 50 μM of Ni(II), Co(II), or Fe(II) ions were added to the electrolyte of 0.25 M KB_i at pH 14 with different sequence, respectively. After the addition of each ion, 20 cyclic voltammetry (CV) scans were performed between 1.01 and 1.71 V vs. RHE with a scan rate of 50 mV s^{-1}.

Supplementary Note to Fig. S5

From the in-situ sequential deposition experiments conducted in Supplementary Fig. S5 the following conclusions can be drawn:

(1) Ni is not the active site in the NiCoFe-B_i catalyst for OER. Regardless of the adding sequence, the CV curves showed negligible change upon addition of Ni(II) ions. The ICP-MS data (Supplementary Fig. S4a) and the deposition potential curve (Supplementary Fig. S4b) for the NiCoFe-B_i catalyst suggest that Ni can be deposited in the potential range of 1.6-1.65 V vs. RHE in the presence of Co(II). Therefore, with CV scans in the range of 1.01-1.71 V vs. RHE, Ni should be incorporated in the catalyst films after the addition of Ni(II) ions for the cases in Supplementary Fig. S5 b, e, and f. However, the CV curves measured after adding Ni(II) ions showed almost no change, which indicates that Ni is not the active site in the NiCoFe-B_i catalyst. Nevertheless, due to their exceptional thermodynamic stability in alkaline conditions, the Ni hydroxides are expected to play an important role in providing a stable host for the Fe active centers.

(2) Fe is the active site in the NiCoFe-B_i catalyst for OER. Upon addition of Fe(II) ions into electrolyte containing Co(II) ions, the OER activity was drastically enhanced, as shown in Supplementary Fig S5 d-f. Although the deposition of only Co hydroxides also showed OER catalytic activity (Supplementary Fig. S5 e-f), the high OER current densities, especially below the potential of ~1.6 V vs. RHE, were achieved after the incorporation of Fe in the catalysts (Supplementary Fig. S5 d-f). This leads to the conclusion that Fe is the active site in the NiCoFe-B_i catalyst, which is consistent with that proposed by Gray *et al.* for NiFe-LDH catalyst[2].

(3) Co catalyzed the electrodeposition of Fe oxyhydroxides. Although Fe is the active site for OER, Supplementary Fig. S5 a-c show that the deposition of Fe oxyhydroxides was not successful in the potential range of 1.01-1.71 V vs. RHE without the presence of Co(II) ions. The high OER activity achieved after adding Co(II) ions suggests Fe was successfully incorporated, which is ascribed to the catalytic effect of Co on the oxidation of $Fe(II)(OH)_2$ to Fe(III)OOH. The catalytic effect of Co bridges the gap between the deposition potential of Fe oxyhydroxides and the operational potential for OER, thus making the self-healing possible.

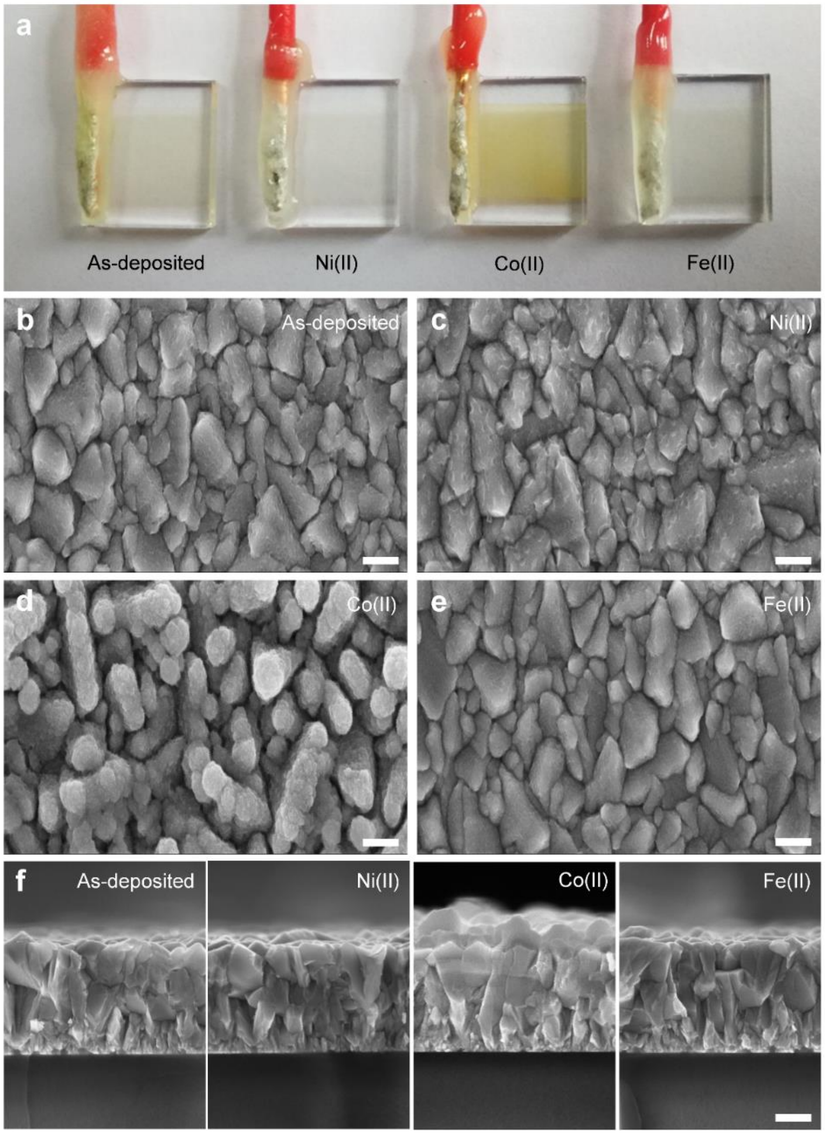

Supplementary Fig. S6 | Morphological changes of the NiCoFe-B$_i$ catalysts after the tests conducted in Fig. 2e. a, Optical image of the NiCoFe-B$_i$ catalyst layers on FTO substrates. **b**, SEM image of the as-deposited NiCoFe-B$_i$ catalyst. **c**, SEM image of the NiCoFe-B$_i$ catalyst after testing in Ni(II)-containing KB$_i$ electrolyte. The significantly increased density of pinholes indicates the dissolution of the catalyst. **d**, SEM image of the NiCoFe-B$_i$ catalyst after testing in Co(II)-containing KB$_i$ electrolyte. The catalyst layer becomes much thicker after the test. **e**, SEM image of the NiCoFe-B$_i$ catalyst after testing in Fe(II)-containing KB$_i$ electrolyte. No obvious morphological change is observed after the test. **f**, Corresponding cross-sectional SEM images of the four samples. The thickening of the catalyst layer after testing in Co(II)-containing KB$_i$ electrolyte can be clearly observed. The scale bars are 200 nm.

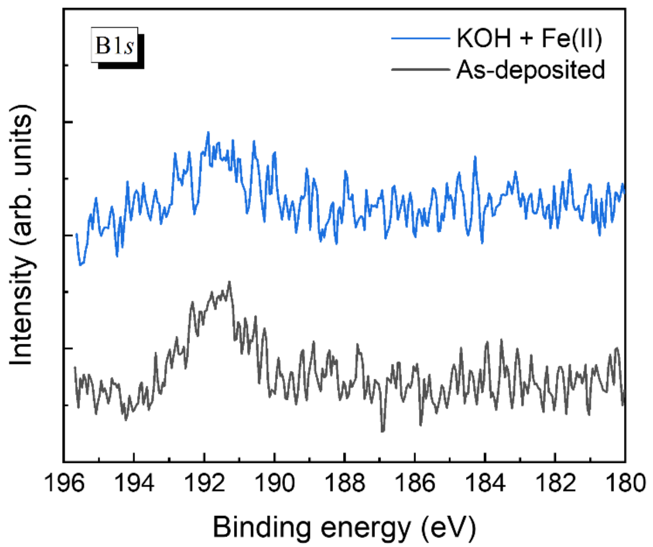

Supplementary Fig. S7 | The B1*s* XPS core level spectra of the NiCoFe-B_i catalyst before and after the stability tests in 1 M KOH electrolyte with 50 μM Fe(II) ions.

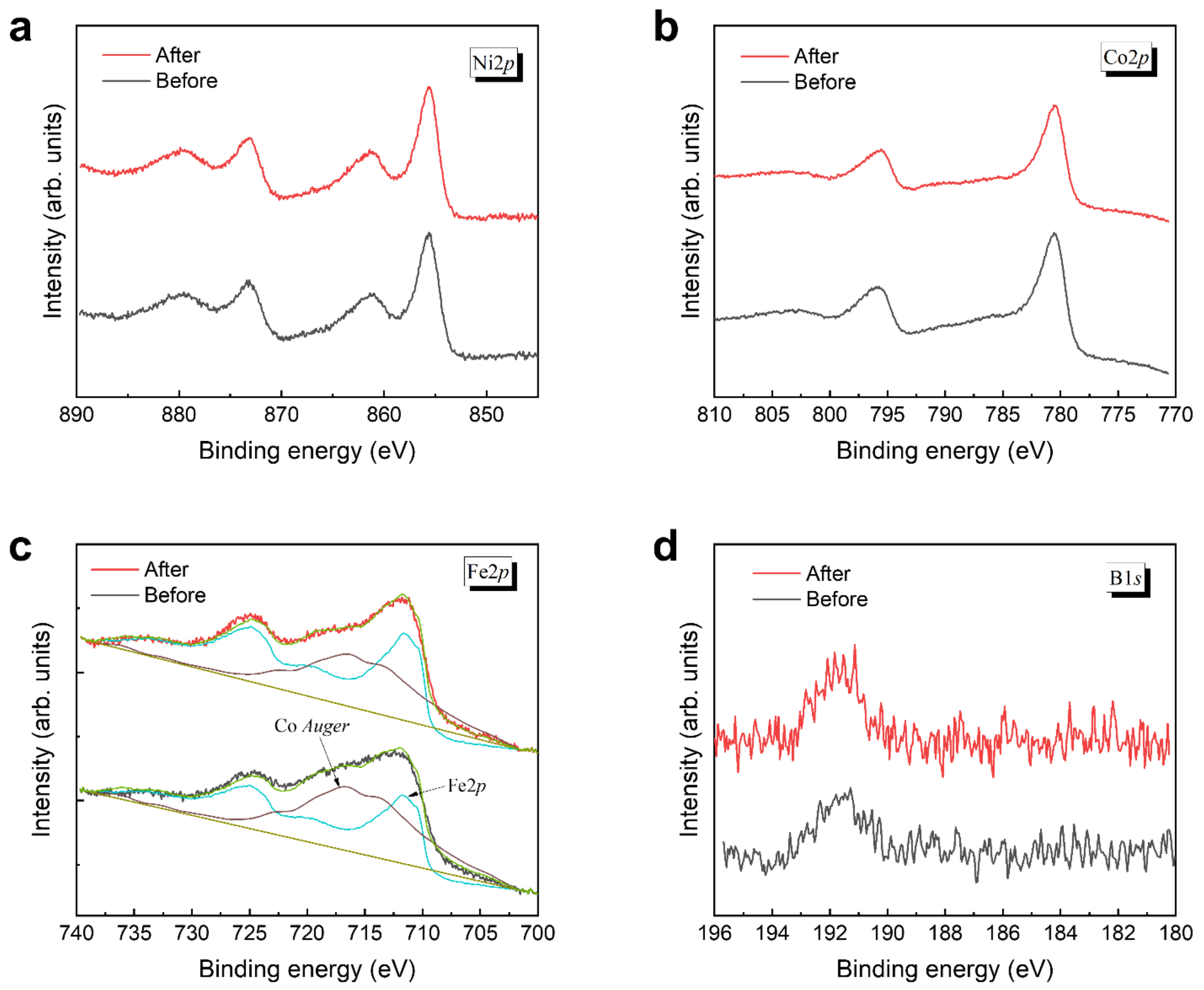

Supplementary Fig. S8 | XPS core level spectra of the NiCoFe-B_i catalyst before and after stability test in KB_i electrolyte at pH 14 with 50 μM Fe(II) ions. **a**, Ni2*p* spectra. **b**, Co2*p* spectra. **c,** Fe2*p* spectra. Due to the overlap of the Co Auger peaks with the Fe2*p* peaks, conventional peak fitting method is not able to distinguish them. Here, the Co *Auger* peaks and Fe2*p* peaks were fitted using the non-linear least squares fitting (NLLSF) function of the Thermo Scientific™ Avantage software. **d**, B1*s* spectra.

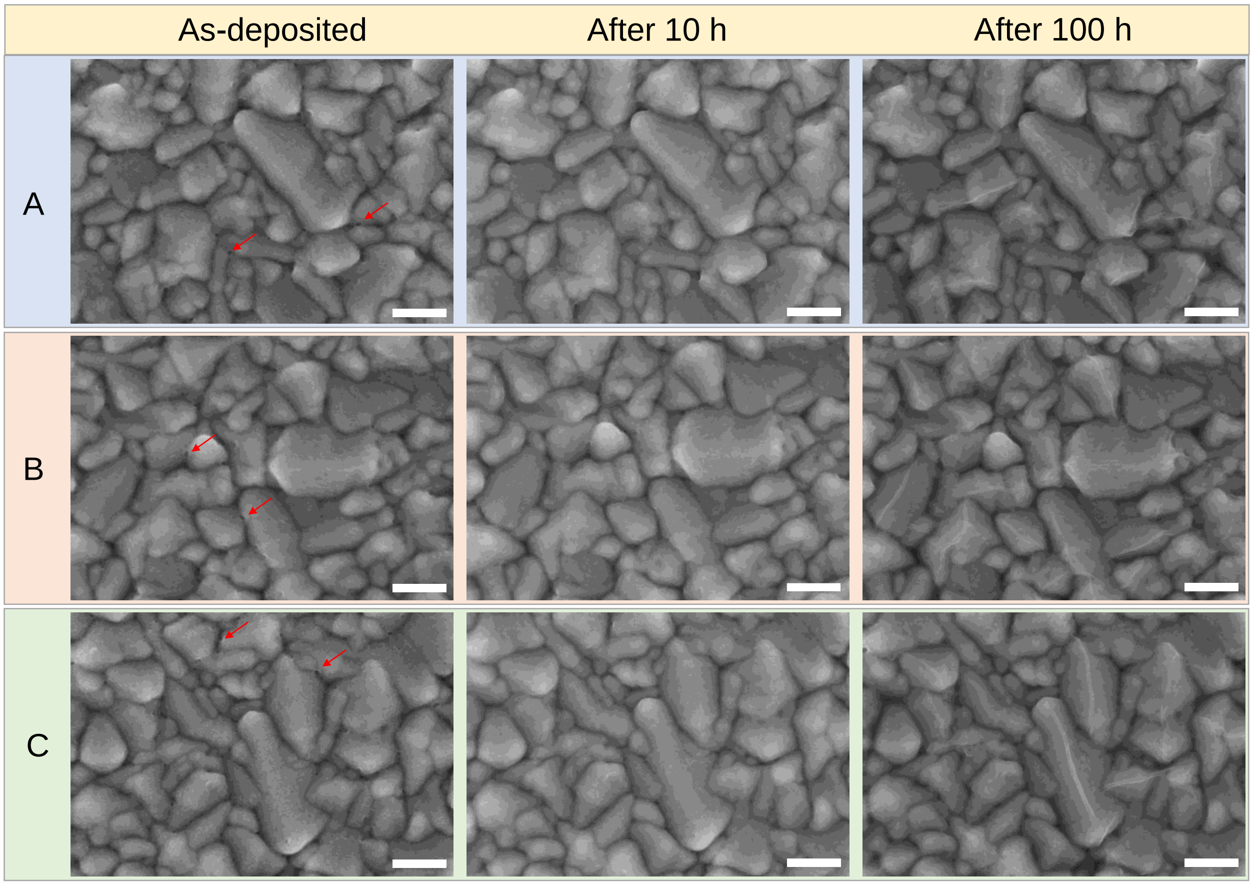

Supplementary Fig. S9 | Morphological change of the NiCoFe-B_i catalyst after stability testing for different times. The stability test was carried out at a constant current density of 10 mA cm^{-2} in KB_i electrolyte (pH 14) with 50 μM Fe(II) ions. Three different locations (A, B, and C) on the same sample were observed with SEM. The scale bars are 200 nm. The as-deposited catalyst covers the grains of the FTO substrate nearly uniformly, except for some small pinholes, as indicated by the red arrows. After testing for 10 h, the pinholes are almost eliminated, indicating the activation of the self-healing mechanism. No pinholes are found after testing for 100 h.

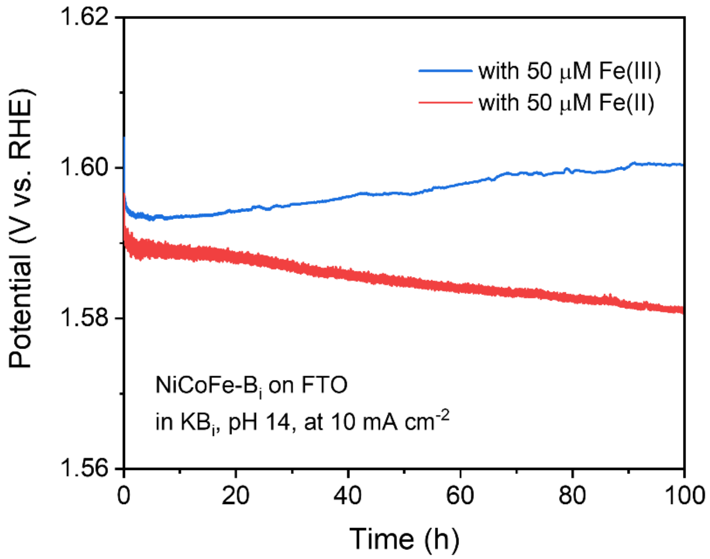

Supplementary Fig. S10 | Chronoamperometry tests of NiCoFe-B_i catalysts on FTO substrate at 10 mA cm^{-2} for 100 h in KB_i electrolytes containing 50 μM Fe(II) or Fe(III) ions.

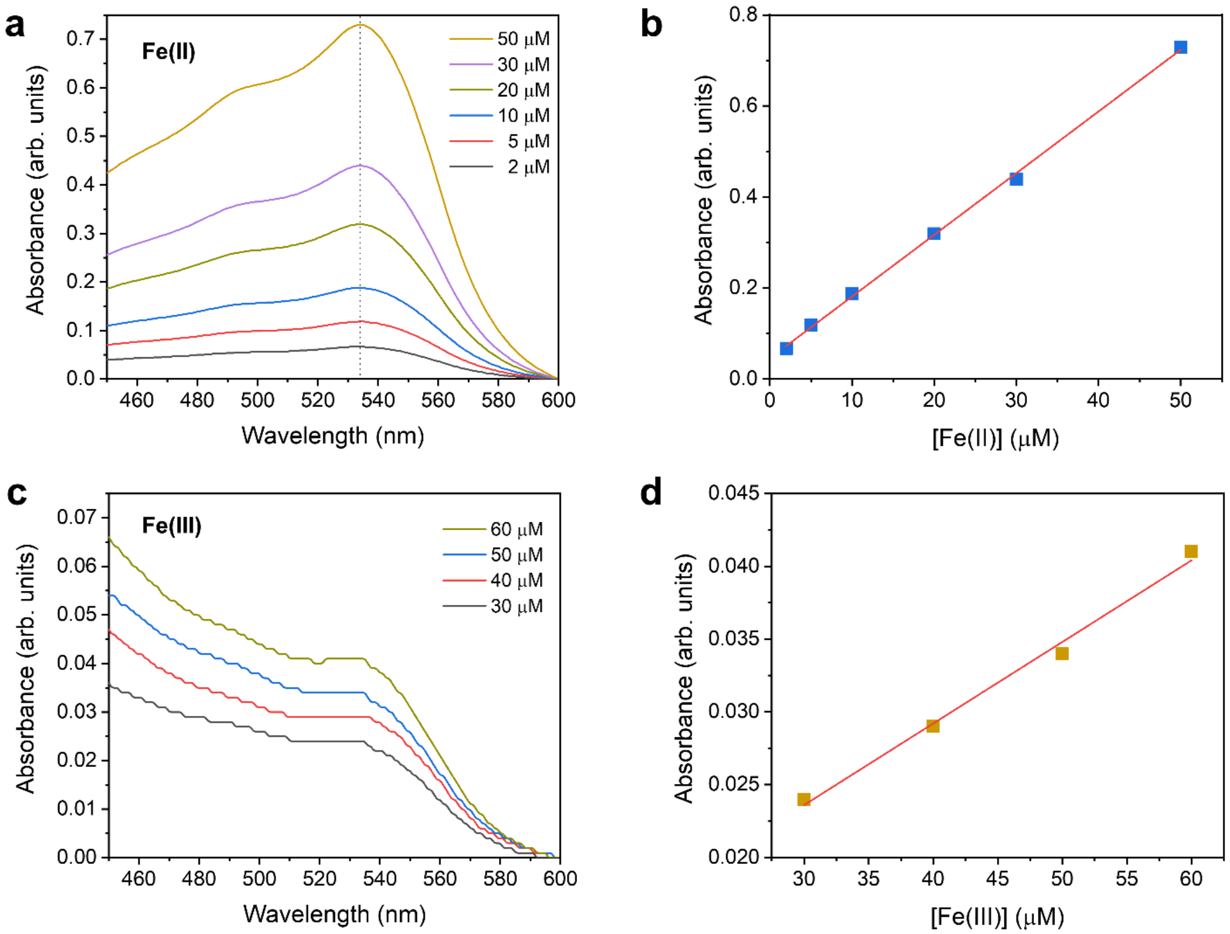

Supplementary Fig. S11 | Calibration curves for the Fe(II) and Fe(III) concentrations in the KB_i electrolyte. a, Absorption spectra of Fe(II)-bathophenanthroline standards with different concentrations. **b**, Plots of the absorbance of the solutions at 533 nm against the Fe(II) concentrations. Calibration curve for the Fe(II) concentrations is obtained from a linear fit (red line) of the data. **c,** Absorption spectra of Fe(III)-bathophenanthroline standards with different concentrations. **d**, Plots of the absorbance of the solutions at 533 nm against the Fe(III) concentrations. Calibration curve for the Fe(III) concentrations is obtained from a linear fit (red line) of the data.

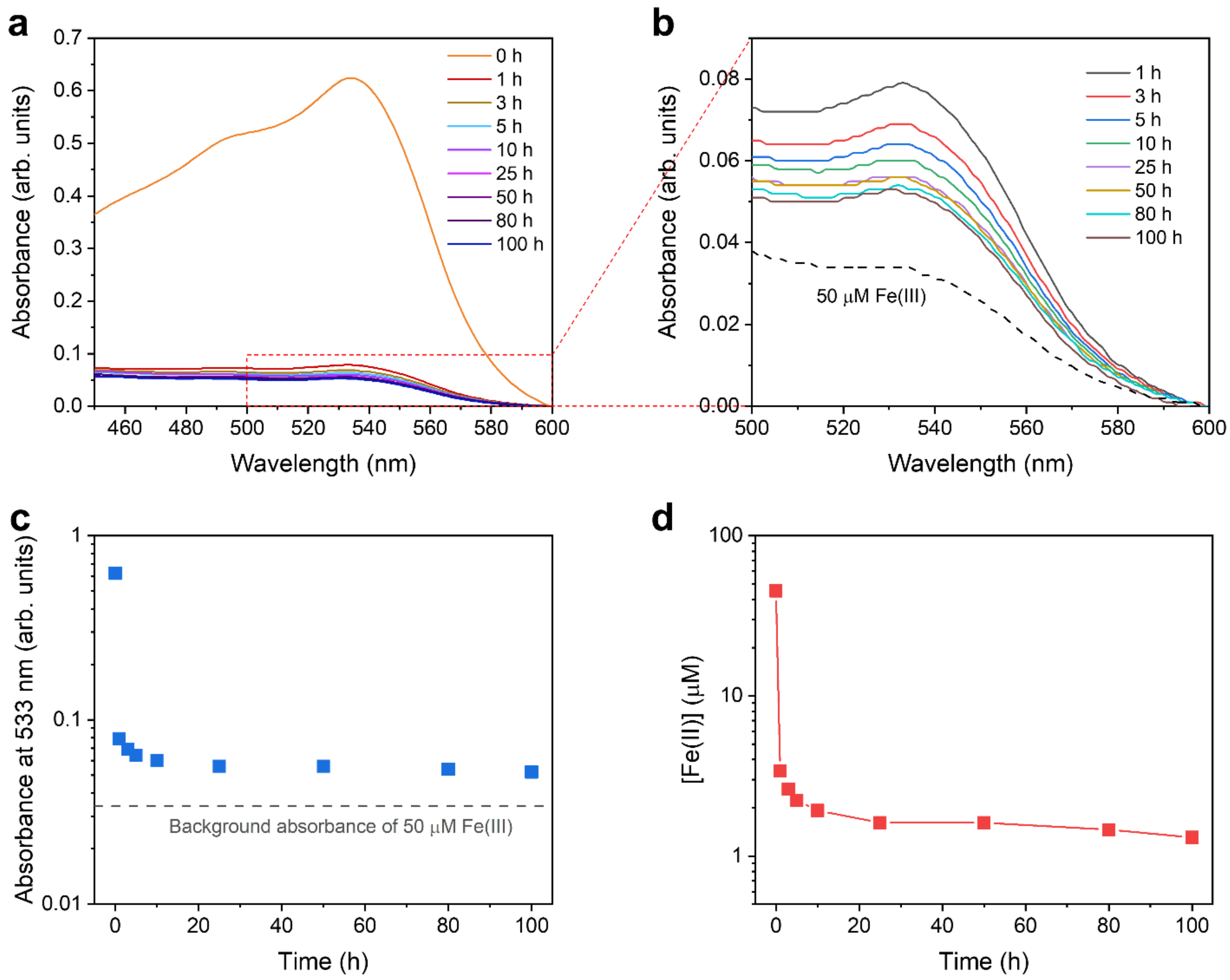

Supplementary Fig. S12 | Determination of Fe(II) concentrations in the KB_i electrolyte during OER test. a, Absorption spectra of samples at different time during the 100-h chronoamperometry test at 10 mA cm^{-2}. **b**, Enlarged view of the absorption spectra of samples taken after 1 h. The background absorption spectrum of the 50 μM Fe(III)-bathophenanthroline solution is also shown by the dashed line. **c,** Change of the absorbance of the samples at 533 nm with time. The background absorbance of the 50 μM Fe(III)-bathophenanthroline solution at 533 nm is shown by the dashed line. **d**, Change of Fe(II) concentrations in the KB_i electrolyte with time.

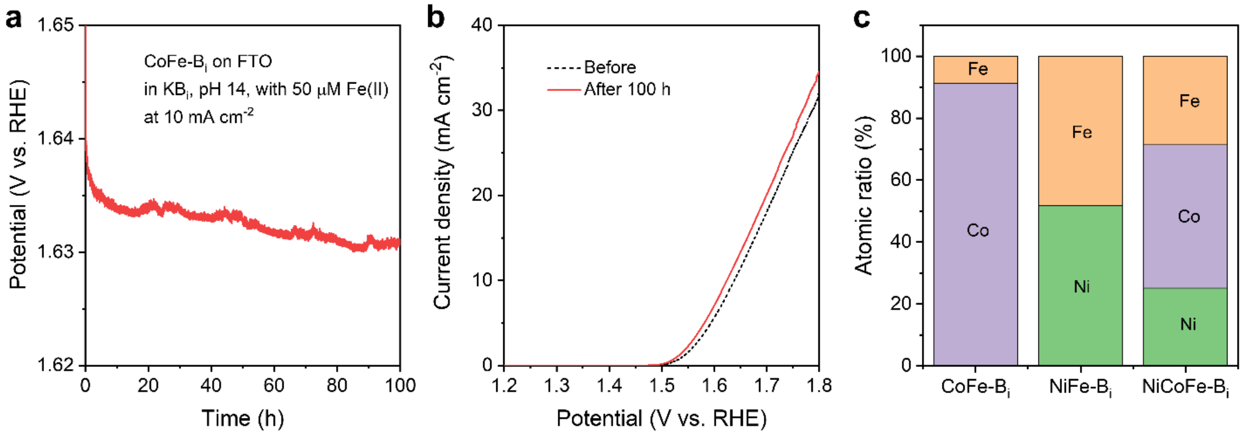

Supplementary Fig. S13 | OER properties of CoFe-B_i catalyst. a, Chronoamperometry test of CoFe-B_i catalyst on FTO substrate at 10 mA cm^{-2} for 100 h in KB_i electrolyte (pH 14) containing 50 μM Fe(II) ions. The CoFe-B_i catalyst was electrodeposited on FTO substrate at a current density of 20 μA cm^{-2} for 8 min. **b,** The OER polarization curves of CoFe-B_i catalyst before and after the stability test. **c**, Atomic ratios of the metal elements in the deposited catalyst layers analyzed by ICP-MS. The Co and Fe molar surface densities of the deposited CoFe-B_i layer were 40.67 and 3.85 nmol cm^{-2}, respectively.

Supplementary Note to Fig. S13

In order to reveal the role of Ni in the catalyst, the activity and stability of a CoFe-B_i catalyst without Ni were tested. Although self-healing was also achieved for the CoFe-B_i catalyst (Supplementary Fig. S13a), the overpotentials were significantly higher (>50 mV) than that of the NiCoFe-B_i catalyst. This is probably because Co hydroxide does not act as an effective host for Fe as compared to Ni hydroxide. As shown by the ICP-MS results in Supplementary Fig. S13c, the atomic ratio of Fe in the deposited CoFe-B_i layer was significantly lower than that in the NiFe-B_i layer. Therefore, even though Ni is not active center for OER, it acts as a stable and effective host for Fe, allowing a high percentage of Fe active centers in the deposited NiCoFe-B_i layer.

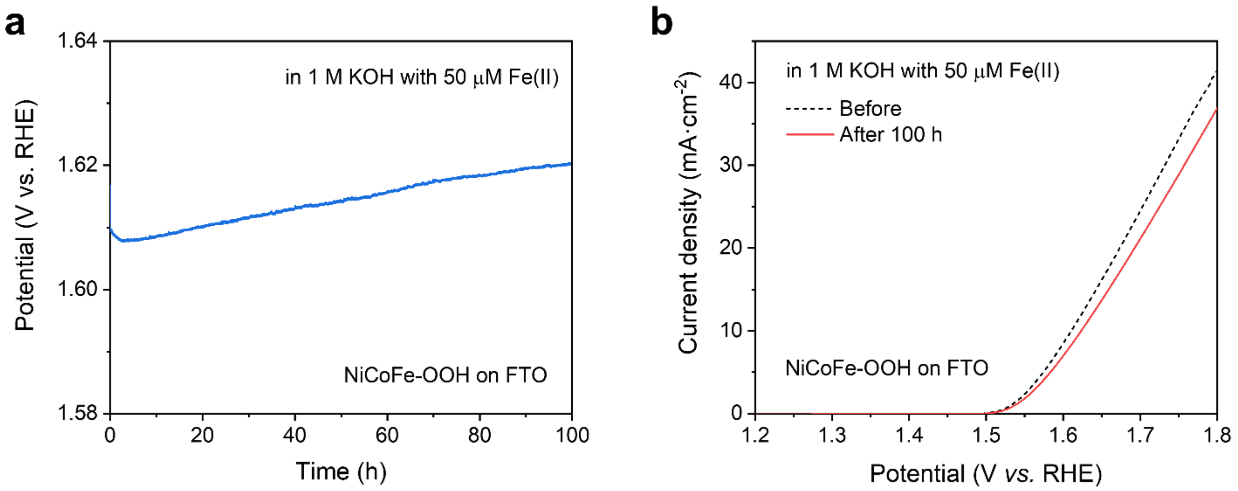

Supplementary Fig. S14 | OER properties of NiCoFe-OOH catalyst. a, Chronoamperometry test of NiCoFe-OOH catalyst on FTO substrate at 10 mA cm^{-2} for 100 h in KB_i electrolyte (pH 14) containing 50 μM Fe(II) ions. **b,** The OER polarization curves of NiCoFe-OOH catalyst before and after the stability test.

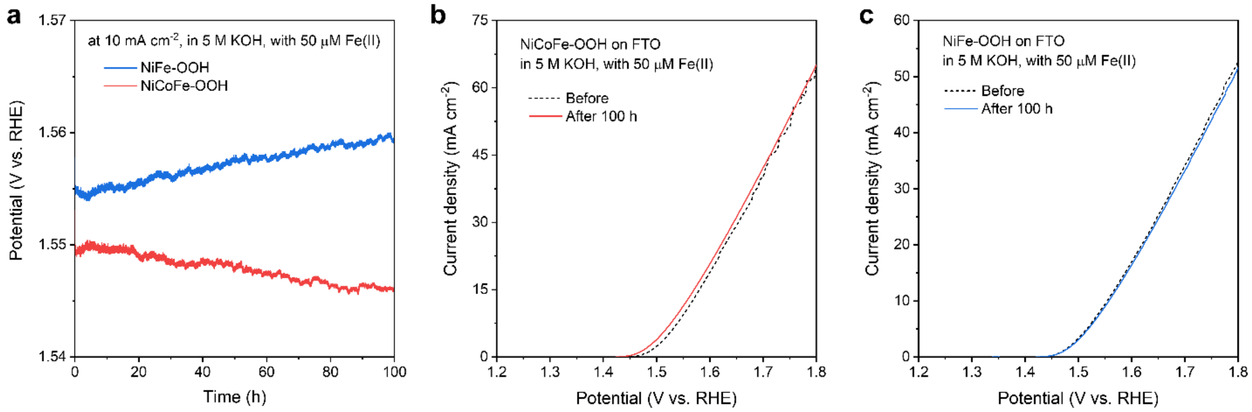

Supplementary Fig. S15 | OER properties of NiCoFe-OOH and NiFe-OOH catalyst. a, Chronoamperometry test of NiCoFe-OOH and NiFe-OOH catalysts on FTO substrates at 10 mA cm^{-2} for 100 h in 5 M KOH electrolyte containing 50 μM Fe(II) ions. **b,** The OER polarization curves of the NiCoFe-OOH catalyst before and after the stability test. **c,** The OER polarization curves of the NiFe-OOH catalyst before and after the stability test.

Supplementary Note to Figs. S14 and S15

In order to clarify the role of borate ions added to the electrolyte, NiCoFe oxyhydroxides (NiCoFe-OOH) catalyst was deposited and tested in electrolytes without borate ions. In detail, the NiCoFe-OOH catalyst was electrodeposited on FTO substrate at a current density of 20 μA cm^{-2} for 8 min in KOH solution at pH 10 containing 0.5 mM $Co(NO_3)_2$, 2 mM $NiSO_4$, and 0.8 mM $FeSO_4$. The NiCoFe-OOH catalyst was then test in 1 M KOH electrolyte with 50 μM Fe(II) ions. (Supplementary Fig. S14). The NiCoFe-OOH showed obvious degradation of OER activity after 100 h. At first glance, one might conclude that the self-healing of the NiCoFe-B_i catalyst is due to the addition of borate ions to the electrolyte. However, as revealed by our ICP-MS results (Supplementary Table S1), the solubility of Fe ions in the electrolyte is significantly enhanced with the addition of borate ions. This should be taken into consideration since the redeposition rate of Fe oxyhydroxide is closely related to the Fe concentration in the electrolyte during the self-healing process. The increased solubility of the Fe ions by the addition of borate ions is likely contribute to the self-healing of the NiCoFe-B_i catalyst in the Fe(II)-containing KB_i electrolyte (Fig. 3b).

As the solubility of Fe ions can also be enhanced by increasing the concentration of the KOH electrolyte[3], we further verified our hypothesis by testing the OER property of the NiCoFe-OOH

catalyst in 5 M KOH electrolyte with 50 μM Fe(II) ions. As shown in Supplementary Fig. S15, self-healing of the NiCoFe-OOH catalyst was indeed achieved in 5 M KOH electrolyte. In contrast, without the presence of Co in the catalyst, the self-healing property is not achieved for NiFe-OOH catalyst in the same electrolyte. From the above results, it is concluded that the borate ions in the electrolyte contributed to the self-healing by increasing the solubility of the Fe ions, while the key to the self-healing is still the catalytic activity of Co on the oxidation of Fe ions.

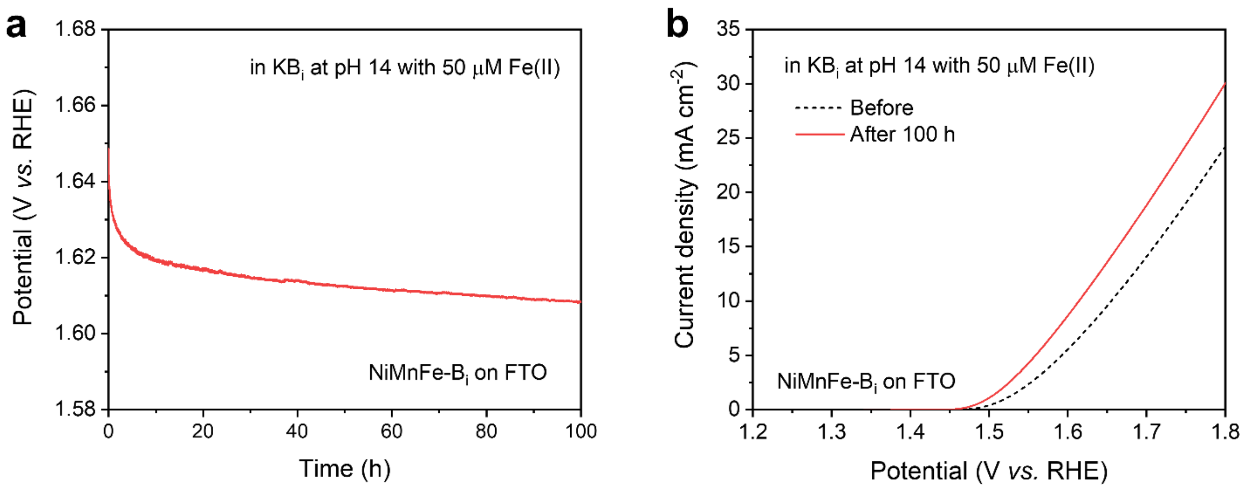

Supplementary Fig. S16 | OER properties of NiMnFe-B$_i$ catalyst. a, Chronoamperometry test of NiMnFe-B$_i$ catalyst on FTO substrate at 10 mA cm^{-2} for 100 h in KB$_i$ electrolyte (pH 14) containing 50 μM Fe(II) ions. **b,** The OER polarization curves of NiMnFe-B$_i$ catalyst before and after the stability test.

Supplementary Note to Fig. S16

According to our proposed self-healing mechanism, the key to achieving self-healing is the catalytic activity of Co on Fe(II)/Fe(III) oxidation for the redeposition of the Fe oxyhydroxide. Since Mn is reported to have similar catalytic activity on Fe(II)/Fe(III) oxidation[1], it should be possible to achieve self-healing by replacing Co with Mn. To demonstrate the universality of our proposed self-healing mechanism, NiMnFe-B$_i$ catalyst was electrodeposited on FTO substrate at a current density of 1 mA cm^{-2} for 8 min in 0.25 M KB$_i$ electrolyte at pH 10 with 0.5 mM $MnCl_2$, 2 mM $NiSO_4$, and 0.8 mM $FeSO_4$. The NiMnFe-B$_i$ catalyst was then tested at 10 mA cm^{-2} for 100 h in KB$_i$ electrolyte (pH 14) containing 50 μM Fe(II) ions. As shown in Supplementary Fig. S16, similar self-healing property was indeed observed for the NiMnFe-B$_i$ catalyst, which demonstrates the redeposition of Fe catalyzed by either Co or Mn is indeed the key to achieving self-healing of the OER catalyst.

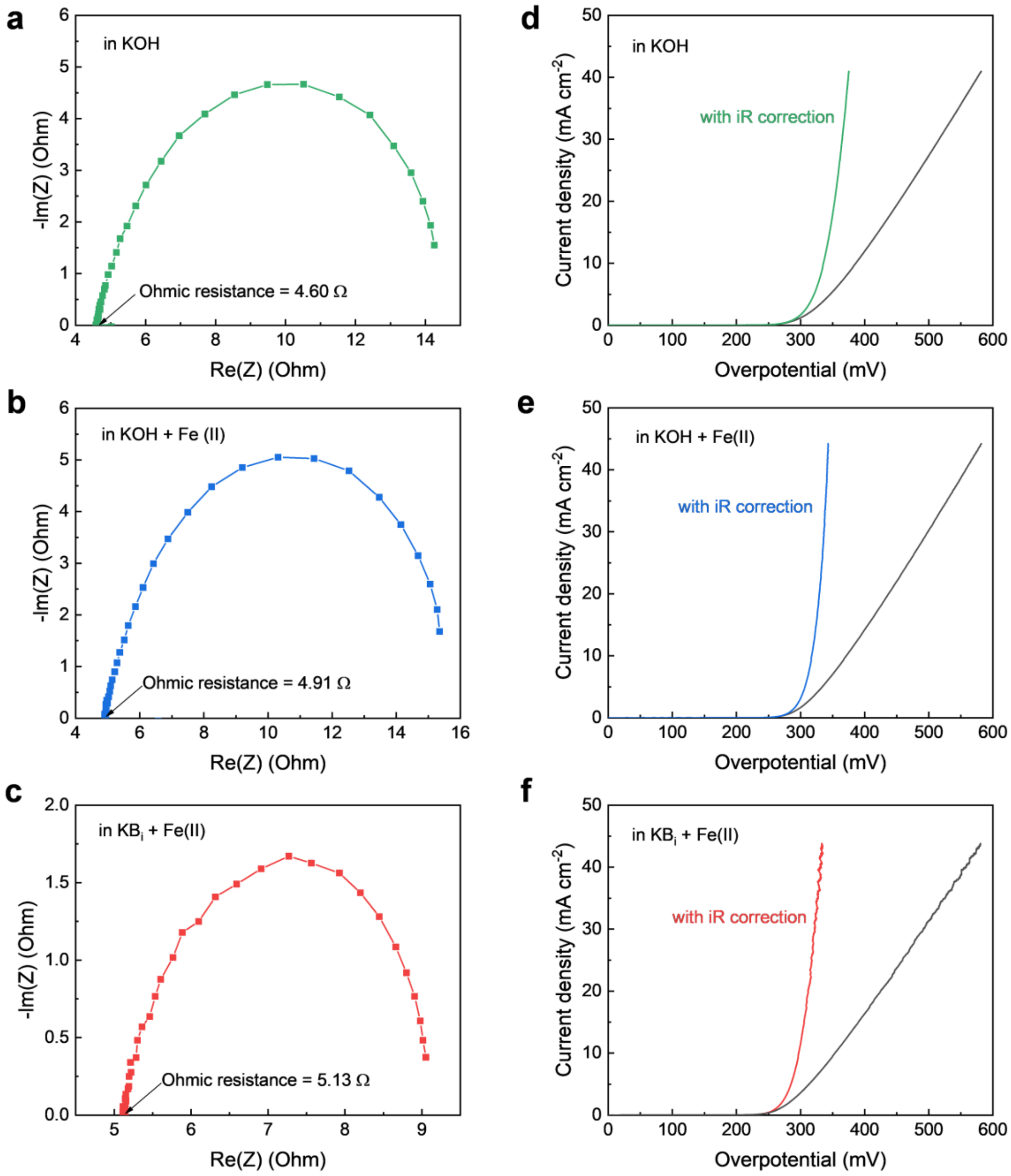

Supplementary Fig. S17 | Ohmic loss (*iR*) correction for the OER polarization curves of NiCoFe-B$_i$ catalysts after stability test in different electrolytes. a-c, Electrochemical impedance spectroscopy (EIS) measured at 1.53 V vs. RHE for the NiCoFe-B$_i$ catalysts in different electrolytes. **d-f,** OER polarization curves with and without iR correction in different electrolytes. Note that the actual measured current (*i*), instead of the current density (*J*), should be used for calculating the *iR* drop.

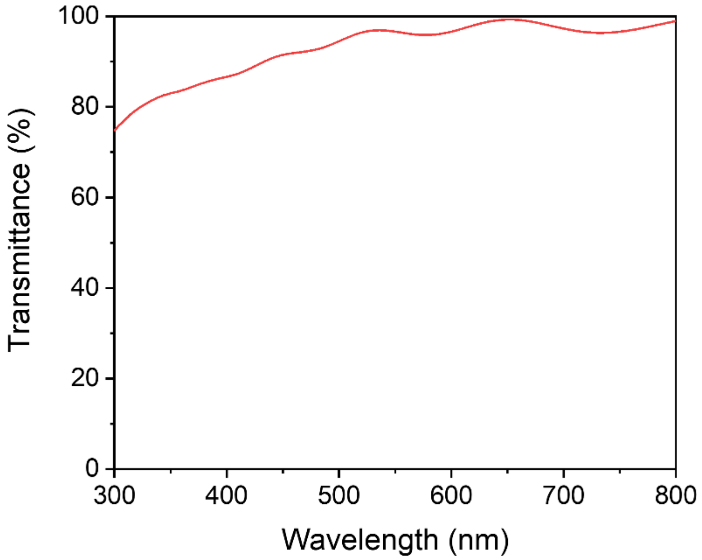

Supplementary Fig. S18 | Transmission spectrum of the NiCoFe-B_i catalyst deposited on FTO substrate. A high transmittance of over ~90% was observed in the visible range.

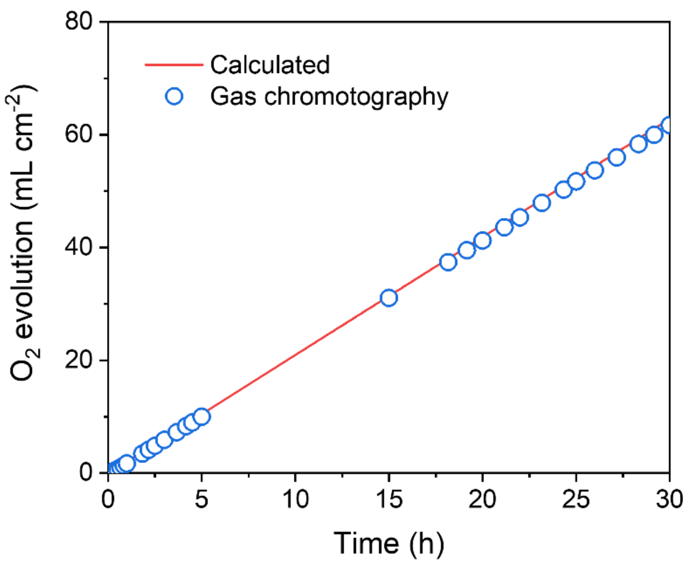

Supplementary Fig. S19 | The amount of evolved O_2 quantified by gas chromatography. The red line indicates the calculated O_2 amount at a current density of 10 mA cm^{-2}, assuming 100% Faradaic efficiency.

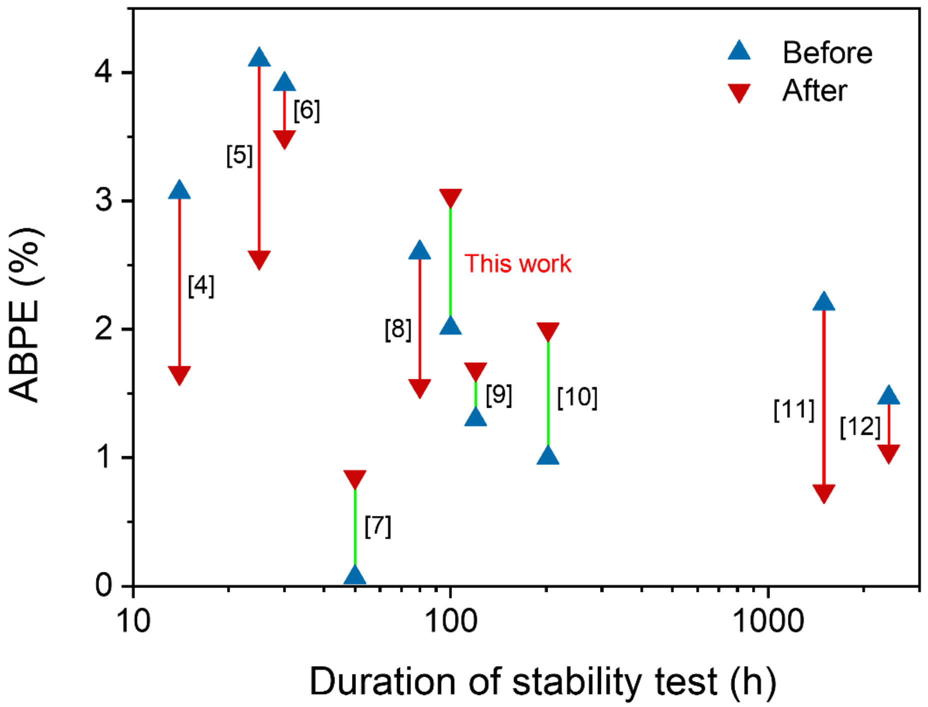

Supplementary Fig. S20 | Efficiency and stability of state-of-the-art n-Si photoanodes[4-12]. The red line indicates a decreasing ABPE during the stability test, while the green line indicates an increasing ABPE during the stability test.

Supplementary Table S1 | Concentration of Fe ions in electrolytes before and after aging. 50 μM Fe(II) ions were added to 1 M KOH electrolyte or 0.25 M KB_i (pH 14) electrolyte. After filtration with 0.2 μm syringe filters, the concentrations of Fe ions in the freshly prepared electrolytes and those aged in air for 100 h were measured by ICP-MS. The ICP-MS results reveal that the solubility of Fe species increases with the presence of borate ions in the KOH electrolyte at the same pH. According to previous study[13], this is attributed to the formation of an aqueous ferrous iron borate complex $[FeB(OH)_4^+]$.

Electrolyte conditions	Fe concentration (μM) measured by ICP-MS	
	Freshly prepared	After 100 h in air
1 M KOH	16.7	2.6
0.25 M KB_i, pH 14	22.7	7.5

Supplementary Table S2 | Roles of each element in the NiCoFe-B_i catalyst. The key species and their thermodynamic stability, and the self-assembly (via oxidation of $M(II)(OH)_2$ to M(III)OOH for M = Ni, Co, Fe) possibility under OER conditions (pH 14, ~1.6 V vs. RHE) are listed.

Elements	Key species	Thermodynamic stability	Self-assembly	Roles
Ni	$Ni(II)(OH)_2$	Stable	No	Stable host
	Ni(III)OOH	Stable		
Co	$Co(II)(OH)_2$	Not stable	Yes	Fe(II) to Fe(III) oxidation catalysis
	Co(III)OOH	Stable		
Fe	$Fe(II)(OH)_2$	Not stable	No	Fe reservoir
	Fe(III)OOH	Stable		OER catalysis
	$Fe(VI)O_4^{2-}$	Not stable	-	
B	$B_4O_5(OH)_4^{2-}$	Not stable	Yes	Improve Fe(II) solubility, improve mass transport

Supplementary References

1. Stumm, W. & Lee, G. F. Oxygenation of ferrous iron. *Ind. Eng. Chem.* **53**, 143-146 (1961).
2. Hunter, B. M. *et al.* Trapping an iron(VI) water-splitting intermediate in nonaqueous media. *Joule* **2**, 747-763 (2018).
3. Ishikawa, K., Yoshioka, T., Sato, T. & Okuwaki, A. Solubility of hematite in LiOH, NaOH and KOH solutions. Hydrometallurgy 45, 129-135 (1997).
4. Liu, Z. *et al.* Tailored NiFe catalyst on silicon photoanode for efficient photoelectrochemical water oxidation. *J. Phys. Chem. C* **124**, 2844-2850 (2020).
5. Moreno-Hernandez, I. A. *et al.* Tin oxide as a protective heterojunction with silicon for efficient photoelectrochemical water oxidation in strongly acidic or alkaline electrolytes. *Adv. Energy Mater.* **8**, 1801155 (2018).
6. Liu, B. *et al.* Bifacial passivation of n-silicon metal–insulator–semiconductor photoelectrodes for efficient oxygen and hydrogen evolution reactions. *Energy Environ. Sci.* **13**, 221-228 (2020).
7. Li, C. *et al.* Highly efficient NiFe nanoparticle decorated Si photoanode for photoelectrochemical water oxidation. *Chem. Mater.* **31**, 171-178 (2018).
8. Luo, Z. *et al.* Multifunctional nickel film protected n-type silicon photoanode with high photovoltage for efficient and stable oxygen evolution reaction. *Small Methods* **3**, 1900212 (2019).
9. Fu, H. J. *et al.* Enhanced stability of silicon for photoelectrochemical water oxidation through self-healing enabled by an alkaline protective electrolyte. *Energy Environ. Sci.* **13**, 4132-4141 (2020).
10. Digdaya, I. A. *et al.* Interfacial engineering of metal-insulator-semiconductor junctions for efficient and stable photoelectrochemical water oxidation. *Nat. Commun.* **8**, 15968 (2017).
11. Zhou, X. *et al.* Interface engineering of the photoelectrochemical performance of Ni-oxide-coated n-Si photoanodes by atomic-layer deposition of ultrathin films of cobalt oxide. *Energy Environ. Sci.* **8**, 2644-2649 (2015).
12. Zhou, X. *et al.* 570 mV photovoltage, stabilized n-Si/CoO_x heterojunction photoanodes fabricated using atomic layer deposition. *Energy Environ. Sci.* **9**, 892-897 (2016).

13. Xiong, Y. *et al.* Experimental determination of stability constant of ferrous iron borate complex [$FeB(OH)_4^+$] at 25 °C from solubility measurements: Implications for transport of iron in reducing environments. *Chem. Geol.* **493**, 16–23 (2018).
